# Personality, Alzheimer's disease and behavioural and cognitive symptoms of dementia: the PACO prospective cohort study protocol

**DOI:** 10.1186/1471-2318-14-110

**Published:** 2014-10-10

**Authors:** Isabelle Rouch, Jean-Michel Dorey, Nawèle Boublay, Marie-Anne Henaff, Florence Dibie-Racoupeau, Zaza Makaroff, Sandrine Harston, Michel Benoit, Marie-Odile Barrellon, Denis Fédérico, Bernard Laurent, Catherine Padovan, Pierre Krolak-Salmon, the PACO group

**Affiliations:** Centre Mémoire de Ressources et de Recherche, Neurology unit, University Hospital of Saint-Etienne, 42055 Saint Etienne, France; Centre Mémoire de Ressources et de Recherche, Geriatry unit, Hôpital des Charpennes, University Hospital of Lyon, 69100 Villeurbanne, France; EA 4607 SNA EPIS – PRES Lyon, Jean Monnet University, 42023 Saint Etienne cedex, France; Centre Hospitalier Le Vinatier, 69677 Bron, France; Hospices Civils de Lyon, Pôle Information Médicale Evaluation Recherche, Université Lyon 1, Equipe d’Accueil 4129, Lyon, F-69424 France; INSERM U1028, CNRS UMR5292, Centre des Neurosciences de Lyon, 69500 Bron, France; Centre Hospitalier Saint Jean de Dieu, Pôle de gérontopsychiatrie, 69800 Lyon, France; University hospital of Bordeaux, Geriatrics Unit, Hôpital Xavier Arnozan, 33604 Pessac cedex, France; Psychiatry Unit, CHU de Nice, Hôpital Pasteur, 06300 Nice, France; CH de Saint Chamond, 42400 Saint Chamond, France; University of Lyon 1, 69500 Lyon, France

**Keywords:** Alzheimer’s disease, Personality, Behaviour

## Abstract

**Background:**

Alzheimer’s disease is characterised by a loss of cognitive function and behavioural problems as set out in the term “Behavioural and Psychological Symptoms of Dementia”. These behavioural symptoms have heavy consequences for the patients and their families.

A greater understanding of behavioural symptoms risk factors would allow better detection of those patients, a better understanding of crisis situations and better management of these patients. Some retrospective studies or simple observations suggested that personality could play a role in the occurrence of behavioural symptoms. Finally, performance in social cognition like facial recognition and perspective taking could be linked to certain personality traits and the subsequent risks of behavioural symptoms.

We propose to clarify this through a prospective, multicentre, multidisciplinary study.

*Main Objective:*

- To assess the effect of personality and life events on the risk of developing behavioural symptoms.

*Secondary Objectives:*

- To evaluate, at the time of inclusion, the connection between personality and performance in social cognition tests;

- To evaluate the correlation between performance in social cognition at inclusion and the risks of occurrence of behavioural symptoms;

- To evaluate the correlation between regional cerebral atrophy, using brain Magnetic Resonance Imaging at baseline, and the risk of behavioural symptoms.

**Methods/Design:**

*Study type and Population*: Prospective multicentre cohort study with 252 patients with Alzheimer’s disease at prodromal or mild dementia stage.

The inclusion period will be of 18 months and the patients will be followed during 18 months. The initial evaluation will include: a clinical and neuropsychological examination, collection of behavioural symptoms data (Neuropsychiatric-Inventory scale) and their risk factors, a personality study using both a dimensional (personality traits) and categorical approach, an inventory of life events, social cognition tests and an Magnetic Resonance Imaging. Patients will be followed every 6 months (clinical examination and collection of behavioural symptoms data and risk factors) during 18 months.

**Discussion:**

This study aims at better identifying the patients with Alzheimer’s disease at high risk of developing behavioural symptoms, to anticipate, detect and quickly treat these disorders and so, prevent serious consequences for the patient and his caregivers.

**Trial registration:**

ClincalTrials.gov: NCT01297140

## Background

Alzheimer’s disease (AD) is characterised by both a progressive cognitive impairment and Behavioural and Psychological Symptoms of Dementia (BPSD) [1]. BPSD are frequent in AD. They may occur at any stage of the disease and progressively worsen as cognitive function declines. Besides the apathy and depression frequently seen in patients with mild to moderate dementia, some of the other BPSD such as agitation, irritability, delusions, aberrant motor behaviour and hallucinations may be extremely upsetting for both the patient and his caregivers [2–4]. When they occur early in the evolution of the disease, their frequency and the intensity of these BPSD can lead to increased difficulties in carrying out everyday tasks [5], an increasingly rapid decline in cognitive function [6], a reduction in the quality of life of both the caregiver and patient [7], an increased likelihood of depression in the caregiver, an increased risk of hospitalisation, premature institution placement [8] and increased health costs [9].

The difficulty in caring for patients with AD is more closely linked to the existence and intensity of the BPSD than the actual cognitive decline. Current treatment for these BPSD is often difficult but is generally approached by medications and/or psycho-social support therapies that still need to be validated.

The prevalence of BPSD has been shown to be increased by a certain number of factors: they may exist at any stage of the disease but become progressively worse as cognitive abilities decline. Evolution of the disease and the level of cognitive impairment have been identified by several authors [4]. A better understanding of the risk factors for the onset of BPSD would allow us to better target those patients at risk, thus favouring earlier treatment and so limiting their consequences on patients, caregivers and the community. The BPSD are the combined result of neurodegenerative lesions [10], somatic comorbidities, iatrogenic effects and also psychological vulnerability. Clinical experience suggests that premorbid personality could play a role in the onset of BPSD with a possible link between personality traits and specific BPSD.

Nevertheless, very few studies have evaluated the relationship between premorbid personality, life events and BPSD. Even childhood trauma could have consequences for the psychosocial adaptation of the elderly [11] and so favour the onset of BPSD [12]. A correlation between a previous history of neuroticism and depression [13, 14] or delusions [15] has been observed in patients with AD. If a high level of social interaction appears linked to an increased risk of agitation, the degree of extraversion does not influence the onset of BPSD [16].

In a previous study, we assessed the influence of previous psychiatric history, premorbid personality and family context on the emergence and typology of BPSD in 99 patients in crisis situations [17]. In this retrospective study, the prevalence of delusions, anxiety, irritability and eating disorders were higher in the patient group with premorbid personality disorders. Those with a history of paranoiac, schizoid or schizotypal problems were more irritable and presented more delusional tendencies. Depression was present in one out of three patients with obsessive-compulsive, avoidant or dependent personality disorders. Finally, eating disorders were more frequent in patients with antisocial, histrionic or narcissistic personalities.

Most of studies assessing the relationship between personality and BPSD are based on the five factors model that defines personality using five dimensions: openness, conscientiousness, extraversion, agreeability and neuroticism. A cross sectional study in 208 patients with moderate to severe Alzheimer’s disease showed an association between a previous history of neuroticism or agreeableness and BPSD, especially for previous neuroticism and anxiety and for previous agreeableness, agitation and irritability [18]. Moreover, a systematic review found a positive relationship between neuroticism and challenging behaviour in 72% of the studies, in particular, mood, aggression and overall challenging behaviour [19]. However, these retrospective studies lacked representative samples and were impacted by confounding factors and flaws in statistical analyses. Finally, a recent study including 52 MCI subjects found a relationship between premorbid neuroticism and openness and affective behavioural symptoms and apathy [20].

Overall, the methodology used in the previous studies makes interpreting results difficult, particularly as most studies are retrospective and include only a small number of patients, often with late stage AD. Such parameters can lead to bias in a patient’s self-evaluation since cognitive decline invariably leads to modification in the ability to express one’s own personality. A second area of bias comes from collecting data retrospectively from relatives. This implies subjective judgements, in an effort to preserve the image of the loved one, it is easy for family to minimise their BPSD or on the contrary to overestimate these behavioural problems and their effects on the family.

Social cognition is a cognitive domain involved in the detection and the recognition of social and emotional messages, as well as integration in a personal emotional context and a social complex context. It concerns one of the major aspects of our cognitive functioning allowing us to interact with our environment. A key component of social cognition is the ability to recognize facial emotion expressions, to identify and to judge mental state and the intentions of the others. It has been shown that impairment in the ability to recognise facial expressions is correlated to social behaviour in various psychiatric and neurological pathologies [21, 22]. Recent work has already shown that patients with AD also had impaired ability to recognise facial expressions [23]. Thus, impairment of social cognition ability in AD could influence the onset and the course of BPSD. Moreover, performance in social cognition tests like facial recognition and perspective taking could be linked to certain personality traits and the subsequent risk of BPSD.

Furthermore, imaging markers could help to understand the link between brain structures changes and the presence of BPSD. The neuropsychiatric symptoms observed in AD patients could be mainly due to frontal structural abnormalities [24]. More specific regional associations with a range of behavioural symptoms have been identified: apathy was associated with frontal structures [25]; delusions were correlated with frontal, parietal, and temporal structures [26]; depressive symptoms with thalamus, lentiform nucleus, and medial temporal cortex [27]; agitation was associated with temporal and frontal structures [28] and amygdale atrophy was related to aberrant motor behaviour, with potential relationships to anxiety and irritability [29]. These same regions participate in the recognition of social messages, particularly those of facial expression, perspective taking and empathy [30]. Consequently, measurement of MRI markers may allow us to better understand the link between personality, social cognition and BDSP and the implication of the anatomic structures in these different domains.

We propose to assess personality in patients with prodromal or mild AD, and then to collect BPSD data prospectively with the objective to evaluate the causality link between personality and BPSD occurrence. Social cognition performance and cortical atrophy will also be measured at baseline.

### Study aim and research question

#### A. Main objective

– To measure the effect of premorbid personality and life events on the likelihood of a patient with probable Alzheimer’s disease on the risk of developing BDSP.

#### B. Secondary objectives

– To evaluate the relationship between premorbid personality and performance in social cognition testing,
– To evaluate the relationship between performance in social cognition testing at study inclusion and the risk of developing BPSD,
– To evaluate the relationship between the distribution of regional cerebral atrophy using 3D brain MRI at study inclusion, social cognition performance and the risk of developing BPSD.

## Methods/design

### Study design

This study is a multicentre prospective cohort study of patients with Alzheimer’s disease. Patients will be followed up over a period of 18 months. At baseline visit, the patients will undergo both a clinical and neuropsychological examination, a study of their personality and important life events, social cognition and blood tests (to screen for exclusion criteria) and a brain MRI. If inclusion is confirmed, the patients will then be followed at 6-month intervals for at least 18 months.

### Setting

The study is being conducted in memory centers of the University hospitals of Lyon, Saint Etienne, Grenoble, Dijon, Bordeaux, Strasbourg, memory centers of Saint Chamond, Annecy and Colmar and Lyon psychiatric hospital.

### Population

*Inclusion criteria are*: diagnostic criteria for AD at mild dementia stage, (NINCDS ADRDA and CDR 1) [31] or prodromal AD (CDR of 0.5) [32], age over 50 years, ability to complete the clinical and neuropsychological evaluations, and presence of a caregiver in sufficient contact with the subject to be able to note the onset of changes in behaviour.

*Exclusion criteria include*: patients with SCPD except from depression, anxiety, apathy, eating or sleep disorders at mild stage, patients with a progressive and/or poorly managed psychiatric pathology (patients with stabilised depression could be included in the study), patients taking *any* neuroleptic psychotropic medication, patients taking other psychotropic medication, with the exception of any antidepressant, hypnotic, anxiolytic, acetylcholinesterase inhibitors or Memantine which has been prescribed and stabilised for more than 3 months, and patients with serious, progressive or unstable pathologies which could interfere with the variables under consideration.

### Informed consent and ethical consideration

Informed written consent is obtained from subject and caregiver before baseline assessment takes place. The study protocol has been reviewed and approved by an ethics committee (Comité de Protection des Personnes Lyon Sud Est III). All procedures are in accordance with the declaration of Helsinki.

### Measures

#### Personality

Personality will be assessed with both categorical and dimensional approaches. The categorical approach looks for personality disorders which are indicative of an underlying psychiatric pathology. The patient will take part in a semi-structured consultation based on the Structural Clinical Interview for DSM-IV Axis II Disorders (SCID II) [33, 34]. The diagnosis will also determine depressive and passive-aggressive personalities included in annexe B of DSM IV. The personality disorder will then be confirmed by collegial decision.

The dimensional approach describes the character traits of each subject. This is done using the NEO PI-R, a widely used questionnaire to assess personality using the « Five-Factor Model » each with 6 facets to each of five major personality types. The « *big five »* model [35] defines 5 basic personality traits: extraversion, openness, agreeableness, neuroticism and conscientiousness.

#### Life events

Major events that may have had an impact on the patient’s future health will be collected using the **Clément questionnaire** [36] and the French **life events scale** designed by Ferreri and Vacher [37], which concentrates on traumatic events in the areas of family life, career, social life, intimate relationships and health. Since the use of these questionnaires has not been validated yet, they will be offered as an additional tool during clinical examination.

#### Assessment of mood

Any current symptomatology indicative of depression will be evaluated using the Geriatric Depression Scale (GDS) which has been validated for use in the elderly [38]. This 30-item scale has proved its usefulness in detecting depressive symptoms in patients presenting with mild dementia. Previous instances of depression will be measured using the MINI [39].

#### Clinical and neuropsychological assessments

An interview, a neurological and general examination will be undertaken by the neurologist, geriatrician or psychiatrist in charge of the patient. Factors favouring BPSD such as family situation, living conditions and intercurrent pathologies will be investigated as will caregiver health using the mini-Zarit test [40].

A standardised battery of neuropsychological tests will be used in the study centres. The Mini Mental State Examination (MMSE) will be used to evaluate global cognitive function [41], the 16-item Free and Cued Recall Test (FCRT) to evaluate verbal episodic memory [42], the Delayed Matching-to-Sample (DMS48) test to evaluate visual recognition memory [43], the Trail Making Test (TMT) [44] and the Stroop test [45], to evaluate executive function, Isaac’s set test to evaluate verbal fluency, the Frontal Assessment Battery (FAB) and the frontotemporal behaviour scale to evaluate dysexecutive behavioural syndrome [46], an 80 item object naming test to evaluate language, a Rey Figure reproduction to evaluate visual construction; meaningless gestures comprehension test assessment will assess praxis. Finally, abilities to perform instrumental activities will be evaluated with the Instrumental Activities of Daily Living (IADL) scale [47, 48].

#### Social cognition

Subjects were engaged in five tasks, including two successive facial feature recognition tasks:

– A test for recognition of facial emotional expression: static colour photographic images of 4 facial emotional expressions (happiness, fear, anger and disgust) were morphed with neutral faces to create an expression continuum. After each face presentation, participants were asked to decide which of five labels (happy, fearful, angry, disgusted, or neutral) best described the presented facial expression using a forced-choice paradigm. Photographs of two female and two male faces depicting basic emotions (happiness, fear, anger and disgust) morphed with a neutral face in 10% steps were randomly presented [21].
– A test for recognition of the facial gender: likewise, photographs of the faces of the 2 sexes (males and women) were morphed with a face depicting “no sex” to create a continuum. The “no sex” was obtained by averaging 20 male and 20 female faces. Eight females and eight males morphed with an average face in 10% steps were randomly presented. Each face had a neutral expression. After each face presentation, participants indicated the gender of the face using a forced-choice paradigm (man or woman) [21].
– A test to detect eye gaze: this uses photographs of the faces of 8 men and 8 women looking 5, 10, 15, 20, 25 or 30° to the right or left. This gives a total of 96 images which are presented in random order. The subject then has to decide whether the person is looking to the right or left [21].
– two tests to assess the subject’s perspective, including: ***a verbal test*** to assess the subject’s ability to describe his own reactions (1^st^ person) and those of his spouse or partner (3^rd^ person) in a variety of social situations presented orally which could be described either as socially neutral or socially emotive [49]; ***a visual test*** to assess the subject’s ability to discern the intentions of others using a short scenario presented as three cartoon drawings. From the subject’s interpretation of the situation and the expected behaviour of one of the characters, he must choose the logical conclusion to the story from among 3 images. A control task uses a scenario that has no character, designed to investigate the subject’s ability to explore and analyse a situation and find a suitable response to it [50].

### Outcome measures: BPSD assessment

Behavioural problems will be assessed during the initial and each subsequent consultation with the patient then by questioning his caregiver. The shortened form of the **Neuropsychiatric Inventory (NPI-Q)** and the **Apathy Inventory** will be used.

The **NPI-Q** is a questionnaire designed to collect informations on neuropsychiatric problems to patients with cerebral disorders. It is an assessment questionnaire administered to the caregiver. It is only designed to evaluate the severity of symptoms and their effect on the caregiver and not their frequency. Twelve types of neuropsychiatric symptoms are covered by the questionnaire: delusions, hallucination, agitation, depression, anxiety, euphoria, apathy, impulsive behaviour, mood swings, aberrant motor behaviour and problems with eating and sleeping.

Symptoms of apathy will also be assessed using the **Apathy Inventory** [51]. **This** provides descriptive assessment of the form this apathy takes, from emotional indifference to lack of initiative and total disinterest in life. The information can be obtained from both the patient and his principal caregiver.

#### MRI

A morphologic MRI will be performed as part of the battery of exploratory tests usually carried out. Acquisition will be performed on a 1.5 Tesla systems. The examination will include a 3D T1-weighted sequence an axial T2 sequence an axial T2* sequence and an axial FLAIR sequence. Voxel Based Morphometry (VBM) procedures will be used to analyze brain volumes and to evaluate the correlation between the score obtained by subjects and the grey matter density on their anatomical MRI scans. The segmentation of 3D T1-weighted images will be performed using SPM2 software and the optimized VBM method described by Senjem et al. [52].

The different measure variables used in PACO study are summarized in Table 1.

**Table 1:** **Measurements instruments used in the PACO study**

***Variable***	***Instrument/source***	***Type of variable***
Demographic variables	Single items	Control variables
Diagnosis variables	NINCDS ADRDA	Control variables
	CDR	
Psychotropic medications	Single items	Control variables
Personality type	NEO PI R	Influencing factor
Personality trouble	SCID 2	Influencing factor
Life events	Clément questionnaire and French Life events Inventory	Influencing factor
Depression	GDS (current depression)	Influencing factor
	MINI (history of depression)	
Neuropsychological tests	Assessment of memory, attention, executive functions, language, praxis and global cognitive functions.	Influencing factor
Social cognition tests	facial emotional expression;	Influencing factor
	recognition of the facial gender;	Secondary outcome variable
	recognition of the facial gender;	
	Theory of Mind tests;	
Neuroimaging measures	MRI Atrophy measurement	Influencing factor
		Secondary outcome variable
Neuropsychiatric symptoms	Neuropsychiatric Inventory (NPI-Q)	Primary outcome measure
Neuropsychiatric symptoms	Apathy Inventory	Primary outcome measure

**Data collection procedures:**

1. *Initial assessment*
  Patients and their caregivers recruited in memory centres will give informed consent. A range of baseline measurements will include clinical interview and examination, assessment of BPSD (NPI and Apathy Inventory). A psychiatric consultation will evaluate previous psychiatric history (MINI), attribution of personality type and/or impairment (NEO PI-R and SCID II) and history of life events. Patients will then undergo neuropsychological assessment and social cognition tests. Biological tests (blood sample) and medical imaging (cerebral IRM) will be done according to usual medical practice.
2. *Follow-up*
  Patients will be followed-up by clinicians at 6 months, 12 months and 18 months. This consultation, besides the standard interview and clinical examination, will include collection of information related to BPSD (NPI and Apathy Inventory) and any factors which may influence behaviour, in particular environment data, caregiver’s burden and concomitant diseases.

**Figure 1:**
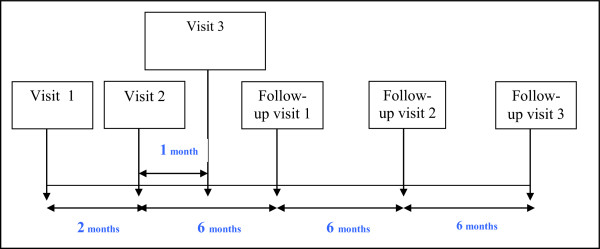
**Typical schedule for a patient enrolled in the PACO protocol: summary of different stages.** Visit 1: clinical visit including assessment of BDSP and their risk factors. Visit 2: psychiatric assessment. Visit 3: neuropsychological assessment and social cognition tests; medical imaging and blood sampling. Follow-up visits: clinical visit including assessment of BDSP and their risk factors.

The different stages of data collection procedure are summarized in Figure 1.

#### Sample size

This has been calculated as a function of our main objective. From the hypothesis that BPSD occurs in 40% of patients [2] with a relative risk (RR) of 1.5 in relation to premorbid personality, the estimated number of subjects is 210 patients for an alpha risk of 5% and a power of 90%. With an expected drop-out rate of 20% over 3 years i.e. 42 patients, the total population required is 252 patients.

#### Data analysis

To assess each study objectives, univariate and then multivariate analyses will be done to take into account potential confusion factors:

To evaluate the link between personality (traits and disorders) and the onset of BPSD, exploratory analyses (e.g. PCA) correlation between personality type or disorder and type of BPSD will be used.

Logistic regression models will be used for binary variables obtained from the NPI-Q and the Apathy Inventory, and linear regression models for continuous explanatory variables: intensity of the behavioural problems and total NPI-Q score.

ANOVA with repeated measures and survival models will be used to study the occurrence of these BPSD over time as seen during the various visits.

Exploratory analyses will be done to define a patient’s typology. Then, correlation between personality type and performance on social cognition tests will be assessed using linear regression models.

The link between baseline performance on social cognition tests and the risk for the onset of BPSD will be assessed with logistic regression models and survival models.

Finally, correlation between the type of BPSD and the difference in distribution of regional cerebral atrophy observed with MRI will be done using specific statistical parametric mapping (SPM) software.

## Discussion

The PACO study aims to better understand the factors underlying neuropsychiatric symptoms in patient with baseline prodromal or mild AD. This prospective multidisciplinary project brings together specialists in old age psychiatry, geriatric and neurology. Its main objective is to evaluate the effect of premorbid personality on the phenomenology and the frequency of BPSD. Premorbid personality will be assessed with the Five Factors Model, which is considered as the most widely used dimensional personality approach [53].

Several studies suggest that personality may influence the behavioral course of AD [19]. It has been yielded that persons with high neuroticism experience have more behavioral disturbances when measured with NPI [18]. The association between personality and specific neuropsychiatric symptoms is so far not fully established and should be better specified. For instance, high levels of neuroticism have been sometimes linked to depression [54] or not [18]. Most studies in this field are retrospective and include few patients, often in advanced stages of the disease. It can induce a substantial risk of recall bias, especially when personality is assessed by informants. The longitudinal design of PACO project, including more than 250 AD patients at prodromal or mild stage, can control some of these biases. Self-report of personality has been preferred to observer rating. In healthy people, this methodology is considered to be more accurate and trustworthy because observers do not have access to a person’s internal motives [55]. In the context of cognitive decline, self-assessment of personality could be criticized. However, Archer et al. showed that the NEO-FFI, a personality questionnaire based on the Big Five Inventory, can be used reliably to measure premorbid personality in AD patients [56]. Moreover, it has been shown that when asked to evaluate their current personality, persons with mild AD tended to describe their former personality [57], maybe due to an inability to update their self-image [58].

In PACO study, we are expecting to find correlations between personality dimensions and the risk of being affected by BPSD. We hypothesize that high level of neuroticism and low level of conscientiousness will be more specifically associated with BPSD, in particular affective domains such as depression, anxiety and apathy. As cognitive decline could be positively or negatively influenced by personality, this potential association will be studied [59, 60]. Moreover, as personality development is considered to be influenced by environmental factors, childhood and adult life events will be collected to assess their possible influence on the course of AD. This would allow us, for example, to highlight the possible effects of previous exposure to a trauma or childhood deficiencies.

Subjects included in PACO study will perform a comprehensive social cognition battery including facial emotion recognition tasks. Several publications have highlighted that AD patients experience an increasing deficit in facial emotional expression recognition as the disease progresses [23], but little is known concerning the very early stage of AD [61]. Consequences of impaired facial emotion recognition on behavioral troubles in AD are questioned and should be clarified [62]. Thus, a secondary objective of the PACO study is to compare social cognition ability between MCI and mild AD, and to identify potential relationships between social cognition abilities and BPSD occurrence during the patient’s follow-up.

PACO project includes MRI 3D sequence at baseline. Different studies have linked impaired emotion recognition in psychiatric or neurologic disease to disturbances or damages of the fronto-temporal brain network [63–65]. Social cognition parameters could be correlated to initial regional cerebral volume and/or the atrophy observed one year later, which could itself be correlated to a higher risk of developing BPSD. For instance, some orbito-frontal atrophy may predict the occurrence of BPSD. Recent evidence suggests that personality traits may be related to neuroanatomical structures. Jackson et al. [66] found that higher neuroticism was associated with specific brain regions atrophy, whereas higher conscientiousness was related to larger regional volumes in healthy aging. It could be hypothesised that an interaction between personality dimension, regional brain atrophy, and BPSD may exist in AD.

The PACO study may have several limitations. Firstly, personality should ideally have been assessed before AD onset. However, it would have been necessary to include a very large number of patients with a long follow-up. We have chosen to include patients at early stage of AD: at this stage we can suppose that the personality assessment reflects premorbid personality. Secondly, a longer follow-up would allow us to detect a larger number of BDSP. The absence of long-term follow-up could underestimate the relationship between personality and BDSP. Indeed, a certain number of SCPD increase with the evolution of the disease, but we hypothesized that some of them occur early in patients with specific personality traits. Thirdly, at baseline only patients with no or mild behavioural disturbances are selected. This inclusion methodology excludes subjects with personality dimensions associated to early behaviour disorder. Finally, the AD diagnosis was based on clinical and MRI criteria. Dubois et al. criteria refer to both imaging and to CSF markers [67]. This would allow better patient selection. However, in our centres, CSF markers are available for a small percentage of the patients, in particular when the diagnosis remains unclear. On the opposite, patients included in the PACO study had a typical clinical presentation, and CSF markers were not available for the majority of them. A systematic lumbar puncture should have limited the feasibility of our study.

The present study also has strengths. In most former studies personality assessment was performed by relatives and only a few included patients at prodromal or mild stage of AD. Moreover, to our knowledge, PACO is the first study to evaluate personality and social cognition performance and their link with subsequent BDSP.

The results of this research may help clinicians to better indentify, from a combined assessment of personality, performance in social cognition tests and the degree of cerebral atrophy, patients with AD at enhanced risk of developing BPSD and to improve patient and family care. Demonstrating a link between premorbid personality and behavioural problems will also allow us to argue for a veritable cooperation between neurologists, geriatricians and psychiatrists in the patients care throughout the course of the disease.

This study also aims to better understand the repercussions of early stage AD on social behaviour, identify the neuroanatomical correlates of BPSD. It should allow us to set up a regional data base to include clinical, neuropsychological, psychiatric, neuroradiological and biological data with longitudinal follow-up of the cohort, and then further inter-regional and inter-speciality cooperation in studying and dealing with Alzheimer’s disease.

## Authors’ information

Isabelle Rouch, MD, PhD, senior epidemiologist, Jean-Michel Dorey, MD, old age psychiatrist, Nawèle Boublay, MSc, epidemiologist, Marie-Anne Henaff, MSc, researcher, Florence Dibie-Racoupeau, MD, old age psychiatrist, Zaza Makaroff, MD, geriatrician, Sandrine Harston, MD, neurologist, Michel Benoit, MD, PhD, old age psychiatrist, Marie-Odile Barrellon, MD, geriatrician, Denis Fédérico, MD, geriatrician, Bernard Laurent, MD, PhD, Head of university neurology unit, Catherine Padovan PhD, neuropsychologist, Pierre Krolak-Salmon, MD, PhD, Head of university geriatric unit.
